# 

**Published:** 1842-05-21

**Authors:** 


**CONTENTS OF No. 21.**

Dr. Neill's Case of Hypertrophy of the Heart and Arteries, - - - 321
Sketches of American Physicians,
Nathaniel Chapman, M. D. - - - - - - - - - 322
BIBLIOGRAPHICAL NOTICES: 1. An Exposition of the Unjust and Injurious Relations of the U. S. Medical Corps. By a Member.
2. Remarks on the Pay of Surgeons of the Navy.
3. Information for Persons desirous of entering the Medical Department of the Navy, - - - - - - - - - 326
Ornithology: Third Book of Natural History, prepared for the use of Schools and Colleges. By W. S. W. Ruschenberger, M. D. Surgeon in the U. S. Navy, &c. - - - - - 333
Dr. E. Hartshorne's Clinical Reports,
Compound Dislocation of the First Phalanx of the Thumb, 333
ANALECTA: Dr. Massey's case of radical cure of Puerperal Ascites, by Spontaneous Exudation through the Parietes of the Abdomen, - - - - - - - - - - - - 334
Dr. Davidson's case of Paracentesis Thoracis, - - - 335

